# Polymerized albumin restores impaired hemodynamics in endotoxemia and polymicrobial sepsis

**DOI:** 10.1038/s41598-021-90431-z

**Published:** 2021-05-25

**Authors:** Donald A. Belcher, Alexander T. Williams, Andre F. Palmer, Pedro Cabrales

**Affiliations:** 1 William G. Lowrie Department of Chemical and Biomolecular Engineering, The Ohio State University, Columbus, OH 43210 USA; 2 Department of Bioengineering, University of California San Diego, La Jolla, CA 92093 USA

**Keywords:** Drug delivery, Biological techniques, Psychology, Medical research, Nanoscience and technology

## Abstract

Fluid resuscitation following severe inflammation-induced hypoperfusion is critical for the restoration of hemodynamics and the prevention of multiorgan dysfunction syndrome during septic shock. Fluid resuscitation with commercially available crystalloid and colloid solutions only provides transient benefits, followed by fluid extravasation and tissue edema through the inflamed endothelium. The increased molecular weight (M.W.) of polymerized human serum albumin (PolyHSA) can limit fluid extravasation, leading to restoration of hemodynamics. In this prospective study, we evaluated how fluid resuscitation with PolyHSA impacts the hemodynamic and immune response in a lipopolysaccharide (LPS) induced endotoxemia mouse model. Additionally, we evaluated fluid resuscitation with PolyHSA in a model of polymicrobial sepsis induced by cecal ligation and puncture (CLP). Resuscitation with PolyHSA attenuated the immune response and improved the maintenance of systemic hemodynamics and restoration of microcirculatory hemodynamics. This decrease in inflammatory immune response and maintenance of vascular wall shear stress likely contributes to the maintenance of vascular integrity following fluid resuscitation with PolyHSA. The sustained restoration of perfusion, decrease in pro-inflammatory immune response, and improved vascular integrity that results from the high M.W. of PolyHSA indicates that a PolyHSA based solution is a potential resuscitation fluid for endotoxic and septic shock.

## Introduction

Sepsis associated mortality and morbidity stem from host–pathogen interactions that can continue long after the initial insult is treated^1^. These interactions lead to systemic inflammatory response syndrome (SIRS), which can result in multiorgan dysfunction syndrome (MODS) if not effectively controlled^2, 3^. Sepsis is the most common cause of non-coronary deaths in intensive care units, and the care and treatment of sepsis costs approximately $20 billion annually in the United States^4, 5^. Proper treatment of sepsis and septic shock has been a controversial research topic due to conflicting results between different pre-clinical models and between different clinical observations^6^. These likely stem from the different etiologies of conditions and infectious agents that precede the insult of sepsis. In addition to controlling and eliminating the initial insult, vasopressor therapies, fluid resuscitation strategies, and the combination of the two, are the most common treatments of sepsis.

**Figure 1:**
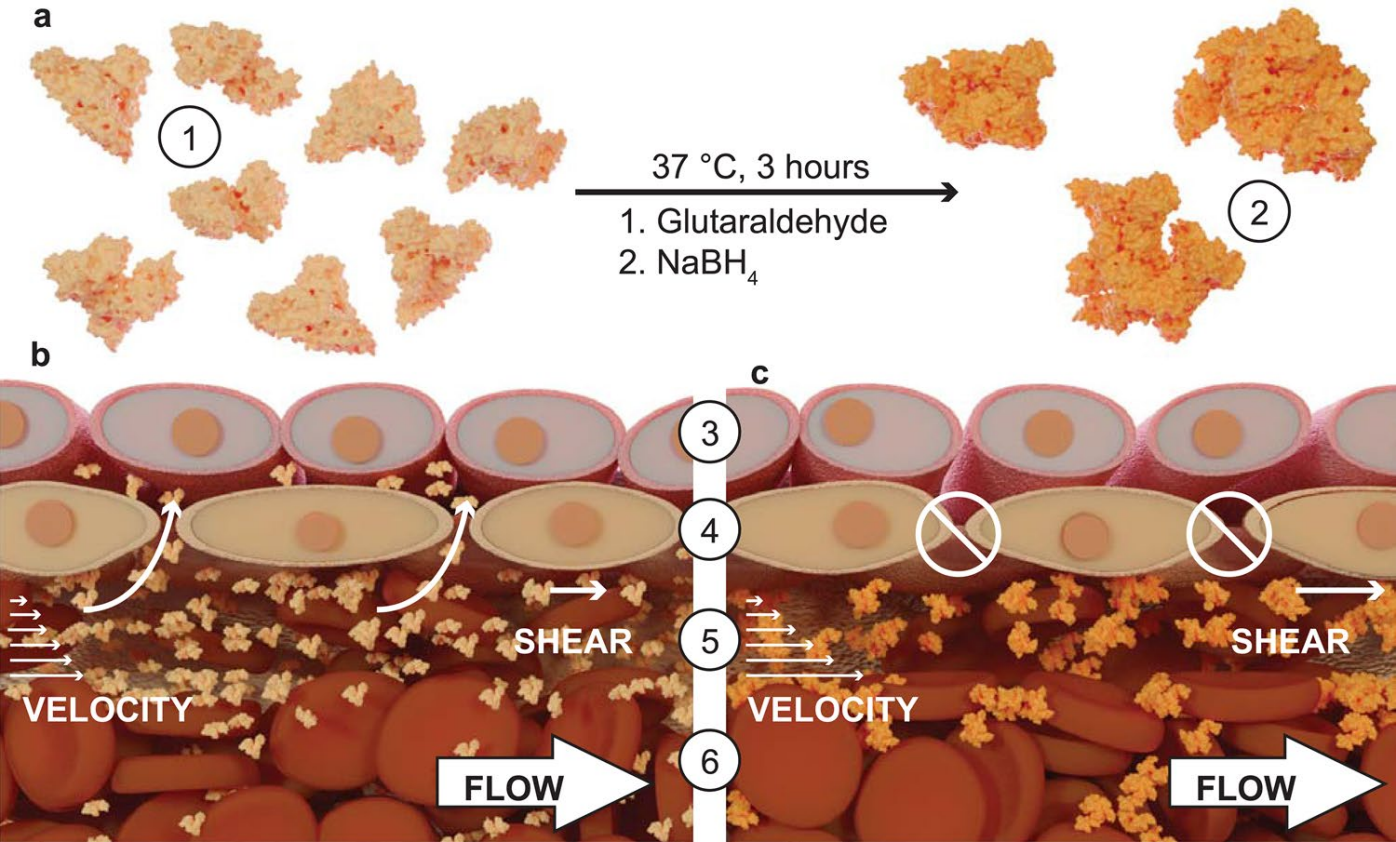
Polymerized human serum albumin synthesis and application as a plasma substitute. (**a**) Human serum albumin (HSA) (1) is reacted with glutaraldehyde to form polymerized HSA (PolyHSA) (2). (**b**) When transfused, unmodified HSA intermingles with red blood cells (RBCs) in the RBC rich core (6) and the RBC depleted plasma layer (5). Due to its small hydrodynamic diameter, HSA is able to easily extravasate through endothelial cell–cell junctions (4) and smooth muscle cell layers (3), which results in reduced circulatory half-life and tissue edema. (**c**) The increased hydrodynamic diameter of PolyHSA leads to increased vascular retention and blood viscosity.

The goal of vasopressor therapy is to maintain blood pressure and flow to vital organs by restricting blood flow to other tissues, such as the skin and gut. Both treatment strategies have been heavily criticized. Some investigators have found that vasopressor therapy can result in impaired gut and sublingual microcirculatory blood flow^7, 8^, while other investigators found no such detriments to the microcirculation^9^. However, restoration of hemodynamic stability via fluid resuscitation is vital to alleviate sepsis-induced hypoperfusion that can result in multiorgan failure^10, 11^. Fluid resuscitation strategies using crystalloids or colloids have been criticized, as they typically only show transient patient benefits, followed by edema and acute respiratory failure. After the initial resuscitation has been completed, septic shock is in part pathophysiologically characterized by deterioration of the vascular endothelial barrier^12^. As a result, small colloidal proteins, such as human serum albumin (HSA), can extravasate from the vascular space into the tissue space, decreasing intravascular, and increasing extravascular colloidal osmotic pressure (COP), which promotes ultrafiltration and prevents reabsorption of fluid (Fig. 1b). As such, a fluid resuscitation therapy that can maintain intravascular oncotic pressure is necessary for the proper care of septic patients.

Hydroxyethylene starch (HES) solutions have been routinely used for fluid resuscitation from septic shock. HES solutions resolve the loss in intravascular COP due to their larger molecular size compared to native colloidal proteins. However, the U.S. Food and Drug Administration recently vetoed the use of HES solutions due to serious adverse effects such as unexpected coagulopathies and renal injury^13, 14^. Previously, we have used polyethylene glycol (PEG) surface conjugated bovine serum albumin (BSA) solutions to improve recovery of functional capillary density (FCD) and tissue oxygenation following lipopolysaccharide (LPS) induced endotoxemia in hamsters^15^. PEGylation significantly increases the molecular size of the BSA molecules, and the hydrophilic nature of PEG increases the oncotic pressure that the molecule can apply. However, the PEGylation process is costly and only increases the COP without increasing the solution viscosity. The cost of PEGylated proteins precludes their use in the generation of plasma expanders from widespread commercialization, and the low solution viscosity of PEG-BSA decreases blood viscosity reducing endothelial shear stress. Alternatively, glutaraldehyde-based protein polymerization is a simple, cost-effective, and scalable strategy to increase the molecular diameter and oncotic pressure of HSA solutions^16–18^. Glutaraldehyde non-specifically reacts with surface proteins to form inter and intramolecular crosslinks between HSA molecules, which can result in significant increases in the effective molecular diameter. A schematic of this process is shown in Fig. 1a. Unlike unmodified HSA, the increased size of glutaraldehyde polymerized HSA (PolyHSA) restricts extravasation (Fig. 1c) and increases solution viscosity, which restores blood viscosity. The increase in intravascular retention, combined with a functional reduction in extravascular COP and an increase in plasma viscosity, restores endothelial shear stress, which may lead to improved maintenance of macro and micro-hemodynamics^17^.

Based on PolyHSA’s ability to maintain blood volume, restore blood viscosity, and hemodynamics, we hypothesize that fluid resuscitation with a PolyHSA solution would improve the maintenance of macro- and micro-circulatory hemodynamics during a controlled septic shock model with systemic inflammation induced by LPS infusion. Endotoxemia was induced via administration of LPS to reduce microvascular blood flow and FCD. Intravital microscopy was used to assess how fluid resuscitation with PolyHSA influences in hemodynamics (vascular tone, blood flow, and FCD). In addition, mean arterial blood pressure (MAP) and heart rate were used as indicators of systemic circulatory response. To assess the impact of fluid therapy on the tissue, endothelial permeability, and cell death (apoptosis and necrosis) were studied in situ in the same tissue where microvascular hemodynamics were observed. Changes in immune response were assessed by monitoring leukocyte interaction in the vasculature and serum cytokine levels. In addition to the LPS model, we also investigated the effects of fluid resuscitation with PolyHSA in a hamster model of polymicrobial sepsis induced by cecal ligation and puncture (CLP).

## Results

### Biophysical properties of PolyHSA

Both formulations were corrected to a total protein concentration of 10 g/dL before transfusion. Polymerization of HSA resulted in decreased COP (18 mm Hg) compared to unmodified HSA (42 mm Hg). Polymerization also resulted in an increase in molecular weight (410 kDa) compared to unmodified HSA (67 kDa) There were no significant changes in viscosity between the PolyHSA (4.2 cP) and unmodified HSA (1.5 cP).

### Endotoxemia systemic hemodynamics

**Figure 2:**
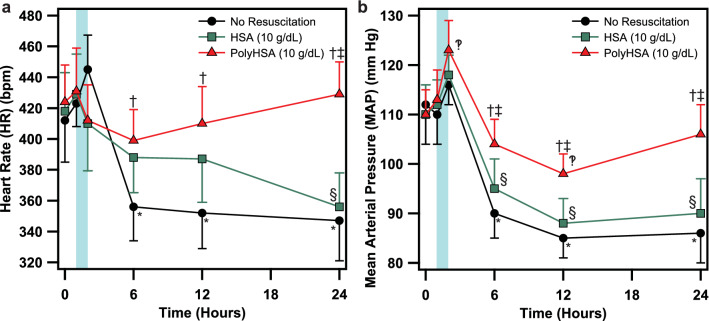
Changes in systemic hemodynamics following LPS induced endotoxemia. (**a**) Heart rate (HR) and (**b**) mean arterial pressure (MAP) with no resuscitation, infusion of HSA, or infusion of PolyHSA. The shaded blue region on the plots indicates the period of fluid resuscitation with PolyHSA or HSA. Data are presented as mean and SD. ^†^P < 0.05 between the PolyHSA and no resuscitation groups at the same time point. ^‡^P < 0.05 between the PolyHSA and HSA groups at the same time point. Symbols next to data points indicate a significant (P < 0.05) difference at that time point compared to baseline conditions for (*) no resuscitation, (§) HSA, and (‽) PolyHSA (n = 6 animals/group).

The changes in HR and MAP during LPS induced endotoxemia are depicted in Fig. 2. All groups had similar HR and MAP at baseline and 1 h following LPS induced endotoxemia. At 2 h after administration of LPS, the MAP was significantly greater in animals resuscitated with PolyHSA compared to baseline conditions. Animals that received no resuscitation had significantly (P < 0.05) decreased HR and MAP compared to baseline conditions 6 h after administration of LPS, which persisted throughout the remainder of the protocol. For animals resuscitated with HSA, there was a significant (P < 0.05) decrease in MAP 6 h after administration of LPS, and MAP did not recover. Additionally, after 24 h, the HR in animals resuscitated with HSA was significantly lower than baseline conditions. There were no other significant differences in HR and MAP compared to the baseline conditions in animals resuscitated with PolyHSA for the entirety of the protocol. From 6 h to the end of the protocol, animals resuscitated with PolyHSA had significantly (P < 0.05) greater MAP compared to animals that received HSA and those that had no fluid resuscitation. At these times, the HR of animals that received PolyHSA was significantly greater than animals that received no fluid resuscitation. Additionally, after 24 h, animals that received PolyHSA had significantly (P < 0.05) greater HR compared to animals resuscitated with HSA.

### Endotoxemia microcirculatory hemodynamics

**Figure 3:**
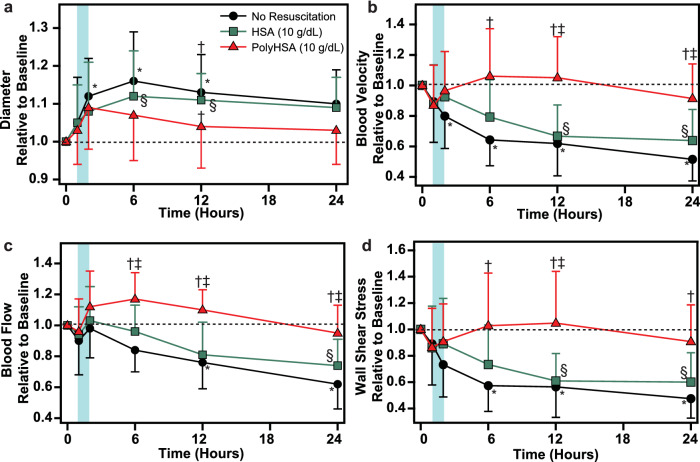
Changes in microcirculatory hemodynamics and wall shear stress following LPS induced endotoxemia. (**a**) Arteriole diameter, (**b**) blood velocity, (**c**) blood flow, and (**d**) arteriole wall shear stress with no resuscitation, infusion of HSA, or infusion of PolyHSA. The shaded blue region on the plots indicates the period of fluid resuscitation with PolyHSA or HSA. Data are presented as mean and SD. ^†^P < 0.05 between the PolyHSA and no resuscitation groups at the same time point. ^‡^P < 0.05 between the PolyHSA and HSA groups at the same time point. Symbols next to data points indicate a significant (P < 0.05) difference at that time point compared to baseline conditions for (*) no resuscitation and (§) HSA (n = 6 animals/group).

Changes in the microcirculatory hemodynamics are shown in Fig. 3. Immediately following LPS induced endotoxemia, the arteriole diameter increased. At the 6- and 12-h mark, animals that did not receive fluid resuscitation and animals the received HSA had significantly (P < 0.05) greater arteriole diameter compared to baseline. Without fluid resuscitation, there was a significant (P < 0.05) decrease in arteriole blood velocity and wall shear stress starting 6 h after LPS administration. In animals that received HSA as the resuscitation fluid, there was a significant (P < 0.05) decrease in blood velocity and wall shear stress compared to baseline conditions 12 h after LPS administration. In these animals, this resulted in significantly (P < 0.05) reduced blood flow at 24 h after LPS administration. For animals resuscitated with PolyHSA, there were no significant changes in arteriole blood velocity and blood flow relative to baseline. Starting 6 h post LPS administration, animals resuscitated with PolyHSA had significantly (P < 0.05) higher arteriole blood velocity and arterial blood flow compared to animals that did not receive fluid resuscitation. Compared to animals that received HSA, the arteriole blood velocity and flow were significantly (P < 0.05) greater in animals resuscitated with PolyHSA at 12 h post LPS administration.

### Endotoxemia functional capillary density

**Figure 4:**
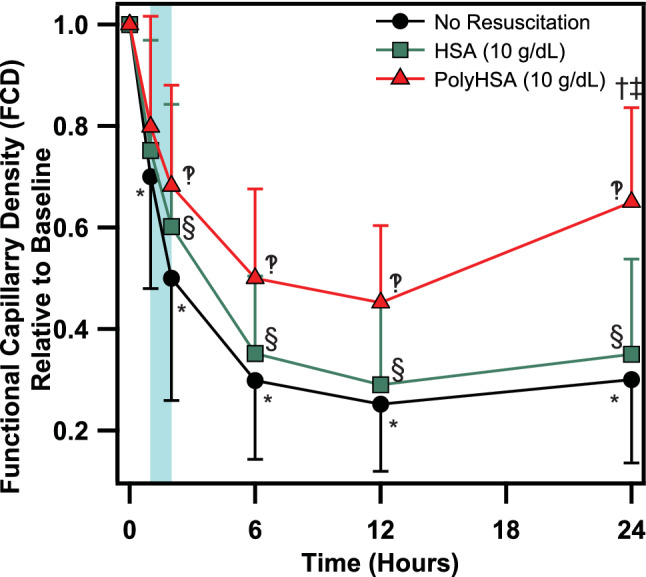
Changes in functional capillary density following LPS induced endotoxemia. Functional capillary density (FCD) with no resuscitation, infusion of HSA, or infusion of PolyHSA. The shaded blue region on the plots indicates the period of fluid resuscitation with PolyHSA or HSA. Data are presented as mean and SD. ^†^P < 0.05 between the PolyHSA and no resuscitation groups at the same time point. ^‡^P < 0.05 between the PolyHSA and HSA groups at the same time point. Symbols next to data points indicate a significant (P < 0.05) difference at that time point compared to baseline conditions for (*) no resuscitation, (§) HSA, and (‽) PolyHSA (n = 6 animals/group).

Changes in the FCD are shown in Fig. 4. In all animals, administration of LPS resulted in significantly decreased FCD compared to baseline conditions. The FCD significantly (P < 0.05) decreased starting 1 h after LPS administration for the no resuscitation treatment groups. Starting 2 h after LPS administration the FCD was significantly lower than baseline conditions in all treatment groups. At the end of 24 h, the FCD in the HSA and no resuscitation treatment groups was still dramatically decreased to ~ 32.5% of the FCD at baseline. In contrast, the FCD was more preserved in animals resuscitated with PolyHSA compared to untreated animals and animals resuscitated with HSA at 6 and 12 h after LPS administration. The FCD significantly (P < 0.05) decreased in the PolyHSA group compared to baseline, and by 24 h the FCD in animals treated with PolyHSA was significantly (P < 0.05) greater than animals in the HSA and no resuscitation treatment groups.

### Endotoxemia leukocyte endothelial interaction

**Figure 5:**
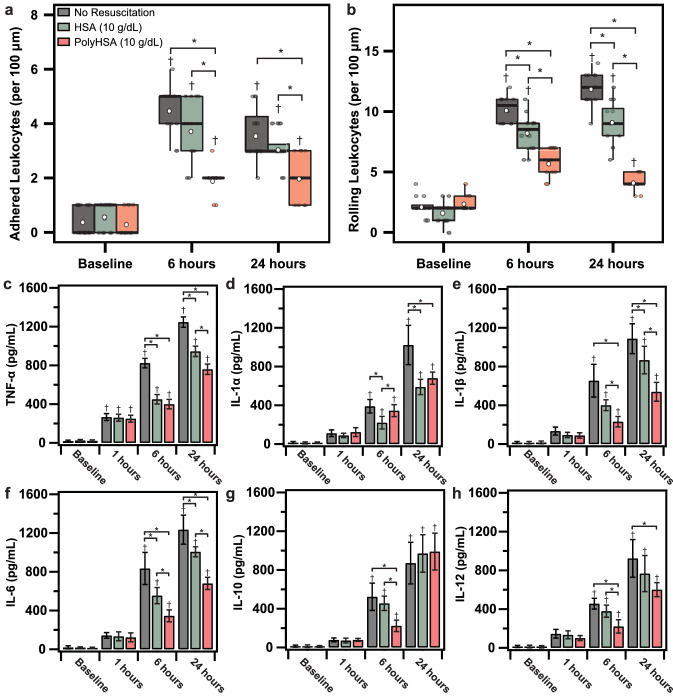
Changes in immune response following LPS induced endotoxemia. Number of (**a**) adhered and (**b**) rolling leukocytes per 100 μm. Increases in serum (**c**) tumor necrosis factor-alpha (TNF-α), (**d**) interleukin 1 alpha (IL-1α), (**e**) interleukin 1 beta (IL-1β), (**f**) interleukin 6 (IL-6), (**g**) interleukin 10 (IL-10), and (**h**) interleukin 12 (IL-12) as measured with ELISA with no resuscitation, infusion of HSA, or infusion of PolyHSA. Data are presented as mean and SD or as boxplots where whiskers indicate 95% CI and boxes indicate data quartiles. *P < 0.05 between groups, ^†^P < 0.05 compared to baseline conditions (n = 6 animals/group).

Changes in the number of leukocytes rolling and adhered to the endothelium is depicted in Fig. 5a,b. At baseline conditions, there were no significant differences in the number of adhered and rolling leukocytes between each treatment group. For all treatment groups, there was a significant (P < 0.05) increase in both adhered and rolling leukocytes after LPS administration. 24 h after resuscitation with HSA, there were significantly fewer adhered and rolling leukocytes compared to animals that did not receive any fluid resuscitation. In animals resuscitated with PolyHSA, the number of both adhered and rolling leukocytes was significantly (P < 0.05) lower compared to the other treatment groups.

### Endotoxemia cytokine ELISA measurements

Changes in serum cytokines tumor necrosis factor-alpha (TNF-α), interleukin 1-alpha (IL-1α), interleukin 1-beta (IL-1β), interleukin 6 (IL-6), interleukin 10 (IL-10), and interleukin 12 (IL-12) as measured with ELISA are shown in Fig. 5c–h. At baseline and immediately prior to fluid resuscitation (1 h after LPS induced endotoxemia), there were no significant differences in cytokines between treatment groups. 1 h after LPS induced endotoxemia, there were significant (P < 0.05) continued increases in serum TNF-α in all treatment groups compared to baseline TNF-α concentration. For all other measured cytokines, there were significant (P < 0.05) increases in cytokine levels at 6 h after LPS induced endotoxemia in each treatment group. For animals resuscitated with HSA and PolyHSA, serum TNF-α and IL-1β levels were significantly lower at 6 and 24 h after LPS induced endotoxemia compared to animals that received no fluid resuscitation. For animals resuscitated with PolyHSA, serum TNF-α and IL-1β levels were significantly lower compared to animals resuscitated with HSA 24 h after LPS induced endotoxemia. 6 h after LPS induced endotoxemia, serum IL-6 levels were significantly lower in animals resuscitated with PolyHSA and HSA compared to animals that underwent no fluid resuscitation. In addition, animals resuscitated with PolyHSA had significantly lower serum IL-6 levels compared to animals treated with HSA 6 h after LPS induced endotoxemia. At 6 h, animals resuscitated with HSA had significantly (P < 0.05) lower levels of serum IL-1α compared to animals that received no fluid resuscitation. At 24 h, the levels of serum IL-1α was significantly (P < 0.05) lower in both the HSA and PolyHSA treatment groups than the group that received no fluid resuscitation.

6 h after LPS induced endotoxemia, animals that underwent fluid resuscitation with PolyHSA had significantly (P < 0.05) lower levels of serum IL-10 compared to the HSA and no resuscitation treatment groups. However, after 24 h, there was no significant difference in levels of serum IL-10 between each treatment group. Animals resuscitated with PolyHSA had significantly (P < 0.05) lower serum IL-12 levels compared to animals that did not undergo fluid resuscitation at 6 and 24 h after LPS induced endotoxemia.

### Endotoxemia apoptosis and necrosis

**Figure 6:**
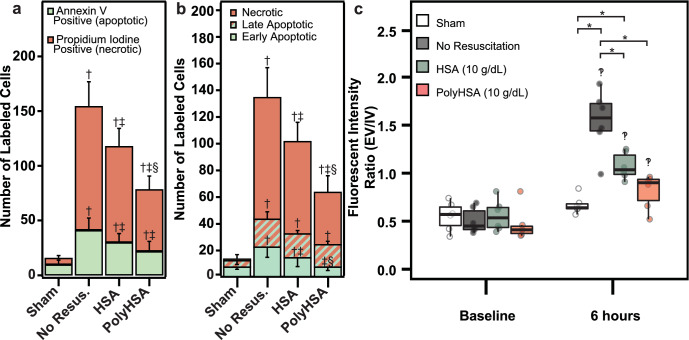
Tissue status following LPS induced endotoxemia. (**a**) Number of annexin V positive and propidium iodine (P.I.) positive stained cells with no resuscitation, infusion of HSA, or infusion of PolyHSA compared to a sham. (**b**) Number of necrotic (PI+/Annexin V−), late apoptotic (PI + /Annexin V +), and early apoptotic (PI−/Annexin V+) cells for each treatment group. (**c**) Endothelial permeability measured via extravascular/intravascular (EV/IV) fluorescent signals from FITC-Dextran (70 kDa M.W.). A higher ratio indicates more vascular leakage. Data are presented as mean and SD or as boxplots where whiskers indicate 95% CI and boxes indicate data quartiles. ^†^P < 0.05 compared to Sham, ^‡^P < 0.05 compared to animals that received no resuscitation, ^§^P < 0.05 compared to animals that received HSA as a resuscitation fluid. *P < 0.05 between groups, ^‽^P < 0.05 compared to baseline conditions (n = 6 animals/group).

The number of cells labeled with Annexin V and propidium iodide (P.I.) and the corresponding count of necrotic (annexin V+/PI−), early apoptotic (annexin V−/PI+), and late apoptotic (annexin V+/PI+) cells 24 h after LPS administration is shown in Fig. 6a,b. For each treatment group, there was a significant (P < 0.05) increase in the number of necrotic cells compared to the Sham control. Tissue from animals that were resuscitated with HSA or PolyHSA had significantly (P < 0.05) fewer necrotic cells than animals that did not receive fluid resuscitation. Additionally, animals resuscitated with PolyHSA had significantly (P < 0.05) fewer necrotic cells than those that received unmodified HSA. Tissue from animals that either received no fluid resuscitation or were treated with HSA had significant (P < 0.05) increases in the number of early apoptotic cells. Whereas tissue from animals resuscitated with PolyHSA had no significant difference in early apoptotic cells compared to the control. Each treatment group had a significant increase in the number of late apoptotic cells compared to the control. However, there were no significant differences between the number of late apoptotic cells between each treatment group.

### Endotoxemia endothelial barrier permeability

The changes in endothelial permeability measured via extravascular/intravascular (EV/IV) fluorescent signals from FITC-Dextran (70 kDa M.W.) extravasation are shown in Fig. 6c. At baseline conditions, there was no significant difference in the intensity ratio between each treatment group. At 6 h after LPS, there was a significant (P < 0.05) increase in the EV/IV intensity ratio compared to baseline conditions for all treatment groups. Animals that underwent no fluid resuscitation or were resuscitated with HSA had significantly greater EV/IV intensity ratios compared to the Sham control. Resuscitation with HSA and PolyHSA resulted in significantly (P < 0.05) decreased EV/IV intensity ratios compared to animals that received no fluid resuscitation. There was no significant difference in EV/IV intensity ratios between the Sham control and animals resuscitated with PolyHSA.

### CLP systemic hemodynamics

**Figure 7:**
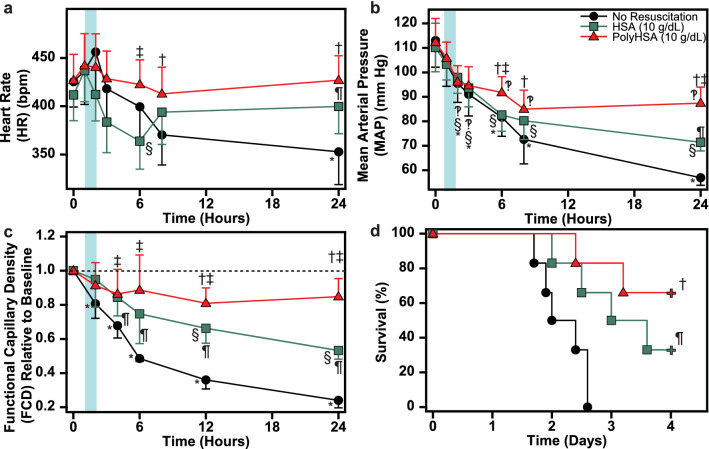
Changes in systemic hemodynamics, functional capillary density and survival following CLP induced polymicrobial sepsis. (**a**) Heart rate, (**b**) mean arterial pressure, (**c**) functional capillary density and (**d**) survival with no resuscitation, infusion of HSA, or infusion of PolyHSA. Data are presented as mean and SD. Survival was assessed via pairwise implementation of the log-rank test. ^†^P < 0.05 between the PolyHSA and no resuscitation groups at the same time point. ^‡^P < 0.05 between the PolyHSA and HSA groups at the same time point. ^¶^P < 0.05 between the HSA and No resuscitation groups at the same time point. Symbols next to data points indicate a significant (P < 0.05) difference at that time point compared to baseline conditions for (*) no resuscitation, (§) HSA, and (‽) PolyHSA (n = 6 animals/group).

Changes in the HR and MAP in animals that underwent the CLP induced septic shock are shown in Fig. 7a,b. At 6 h following the CLP procedure, the HR in the HSA treatment group was significantly lower than baseline conditions and animals in the PolyHSA treatment group at the same time point. At 24 h, the HR of animals in the HSA treatment group was significantly higher than animals in the no resuscitation group. After 8 h, animals that received PolyHSA had significantly higher HR than animals that received no fluid resuscitation. Starting 2 h after the CLP procedure, there was a significant (P < 0.05) sustained decrease in MAP. After 6 h, animals in the PolyHSA treatment group had significantly (P < 0.05) higher MAP compared to the no resuscitation group. At 6 and 24 h, animals in the HSA treatment group had significantly lower MAP compared to animals in the PolyHSA treatment group.

### CLP microcirculatory hemodynamics

**Figure 8:**
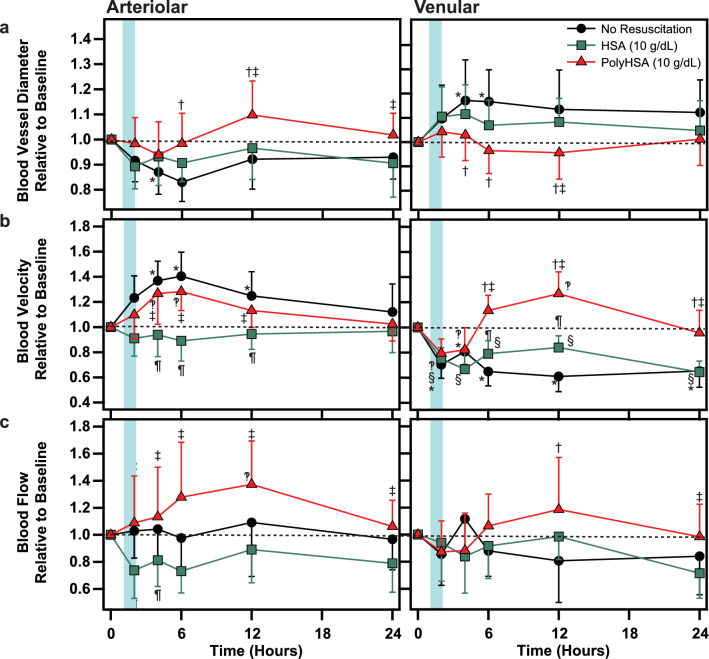
Changes in microhemodynamics following CLP induced polymicrobial sepsis. Arteriolar and venular (**a**) blood vessel diameter, (**b**) blood fluid velocity, and (**c**) blood flow with no resuscitation, infusion of HSA, or infusion of PolyHSA. ^†^P < 0.05 between the PolyHSA and no resuscitation groups at the same time point. ^‡^P < 0.05 between the PolyHSA and HSA groups at the same time point. ^¶^P < 0.05 between the HSA and no resuscitation groups at the same time point. Symbols next to data points indicate a significant (P < 0.05) difference at that time point compared to baseline conditions for (*) no resuscitation, (§) HSA, and (‽) PolyHSA (n = 6 animals/group).

Changes in the microcirculatory hemodynamics in animals that underwent CLP induced septic shock are shown in Fig. 8. At 6 and 12 h, the relative arteriolar diameter was significantly (P < 0.05) expanded in animals resuscitated with PolyHSA compared to animals that received no fluid resuscitation. After 12 h, the relative arteriole diameter in animals in the HSA treatment group was significantly lower (P < 0.05) than animals in the PolyHSA treatment group. Between 2 and 12 h, the arteriolar blood velocity in animals resuscitated with HSA was significantly lower (P > 0.05) than animals that received no fluid resuscitation or animals resuscitated with PolyHSA. In animals in the no resuscitation and PolyHSA treatment group, there was a significant increase in blood velocity compared to baseline conditions. Beginning 2 h after fluid resuscitation, there was a significant (P < 0.05) increase in arteriolar blood flow in animals in the PolyHSA treatment group compared to animals in the HSA treatment group.

At 4 and 6 h, animals that received no fluid resuscitation had significantly (P < 0.05) higher venular blood vessel diameter compared to baseline conditions and animals in the PolyHSA treatment group at the same time point. In all animals, there was a significant (P < 0.05) decrease in the venular blood velocity at 2 and 4 h compared to baseline conditions. This decrease in venular blood velocity was sustained for the remainder of the observation window in animals that received no fluid resuscitation and animals that received HSA. Starting at 6 h, the venular blood velocity in the PolyHSA treatment group was significantly (P < 0.05) greater compared to animals in the HSA and no resuscitation treatment group.

### CLP functional capillary density

Changes in the FCD in animals that underwent CLP induced septic shock are shown in Fig. 7c. In animals that received no fluid resuscitation, there was an immediate and sustained (P < 0.05) decrease in FCD compared to baseline conditions. Beginning 4 h after fluid resuscitation, the FCD in animals in the HSA and PolyHSA groups were significantly (P < 0.05) greater compared to animals that received no resuscitation. At 12 and 24 h following fluid resuscitation, animals in the HSA treatment group had significantly (P < 0.05) lower FCD compared to baseline conditions and animals in the PolyHSA group at the same timepoint.

### CLP survival

The survival curves for animals that underwent CLP induced septic shock are shown in Fig. 7d. Resuscitation with both HSA and PolyHSA significantly (P < 0.05) improved mean survival time compared to animals that received no fluid resuscitation. At four days following CLP, twice the number of animals resuscitated with PolyHSA survived compared to animals in the HSA treatment group.

## Discussion

The principal finding of this study was that fluid resuscitation with PolyHSA restores impaired microvascular function after LPS induced endotoxemia and CLP induced polymicrobial sepsis. Overall, fluid resuscitation with the PolyHSA solution resulted in increased normalization of MAP, HR, FCD, and microcirculatory blood flow. When compared to resuscitation with standard HSA, fluid resuscitation with PolyHSA resulted in significantly improved restoration of systemic hemodynamics, microcirculatory hemodynamics, and vascular permeability. Despite observing increases in arteriole diameter and blood flow following resuscitation with PolyHSA, we still observed significant decreases in FCD compared to baseline conditions in LPS induced endotoxemia, but the loss of FCD was attenuated compared to animals that received no fluid resuscitation or unmodified HSA. Unlike arteriole diameter and blood flow, FCD begins to decrease immediately as endotoxemia starts following IV administration of LPS. The continued loss of FCD is likely a result of sustained damage to the endothelial barrier resulting in increased extravascular hydrostatic pressure from extravasation of colloidal proteins. However, in animals resuscitated with PolyHSA, we observed significantly reduced endothelial permeability compared to other treatment groups. Extravasation of PolyHSA is reduced by its increased molecular size, which results in improved maintenance of blood volume, MAP, and capillary pressure, thus preserving FCD. Unlike in LPS induced endotoxemia, animals that underwent CLP induced polymicrobial sepsis had a much slower decay in FCD for the corresponding treatment groups. At around 12 h, the change in FCD was comparable between animals that underwent LPS induced endotoxemia and CLP induced polymicrobial sepsis. These significant improvements in FCD following resuscitation with PolyHSA in CLP induced polymicrobial sepsis is likely what leads to the improved survival in the PolyHSA treatment group.

Fluid resuscitation with PolyHSA helps diminish the overactive inflammatory immune response in LPS induced endotoxemia. LPS is recognized by toll-like receptor 4 (TLR4) in all cell-types^19^. This leads to a complex inflammatory cascade, and one of the many consequences of this cascade is the classical activation of macrophages (M1 macrophages). These M1 macrophages release TNF-α and other inflammatory cytokines. As we observed in this study, resuscitation with PolyHSA significantly reduced the innate immune response to LPS, evidenced by a significant reduction in pro-inflammatory cytokines. The decrease in pro-apoptotic cytokines (IL-1β, TNF-α) in animals resuscitated with PolyHSA may contribute to the decreased apoptotic cell fraction observed in the tissue. Despite observing decreases in the early-apoptotic cells in the PolyHSA and HSA resuscitation treatment groups, the number of late apoptotic cells was consistent across all groups. The consistent presence of this group of cells likely results from the initial pro-inflammatory response and decreased capillary perfusion. Taken together, these changes in the apoptotic cell fractions indicate a superior recovery in the tissues following resuscitation with PolyHSA.

While resuscitation with PolyHSA did not entirely ameliorate leukocyte adhesion to the endothelium, we still observed significant improvements compared to the no resuscitation and HSA resuscitation groups. This is strong evidence of the preservation of the glycocalyx in animals resuscitated with PolyHSA, as the glycocalyx regulates neutrophil adhesion^20^. Despite observing decreased expression of the anti-inflammatory cytokine, IL-10, all measured pro-inflammatory cytokines were suppressed in animals resuscitated with PolyHSA. This temporary decrease in anti-inflammatory responses indicates that resuscitation with PolyHSA may help attenuate the intensity of the initial immune response. 24 h after LPS administration, anti-inflammatory cytokines were normalized across each treatment group. This restoration of IL-10 at 24 h indicates that resuscitation with PolyHSA does not have a long-term effect on suppressing the anti-inflammatory response.

Overall, fluid resuscitation with PolyHSA resulted in decreases in pro-inflammatory cytokines compared to resuscitation with HSA and no fluid resuscitation. This decrease in pro-inflammatory response may contribute to the improved vascular integrity in the PolyHSA treatment group. Without proper treatment, the immune response, mainly driven by TNF-α, causes the release of reactive oxygen species (ROS), ultimately damaging endothelial cells^21^ and causing glycocalyx shedding^22–24^. The endothelial glycocalyx plays a vital role in retaining intravascular oncotic pressure by blocking negatively charged proteins, such as HSA, from passing between endothelial cells. HSA flowing into the extravascular area may worsen edema by increasing extravascular oncotic pressure.

Although the decreases in inflammatory cytokines resulting from PolyHSA resuscitation may have an impact on glycocalyx integrity, the increased wall shear stress in the PolyHSA treatment group may facilitate increased glycocalyx regeneration following LPS-induced endotoxemia. Recent studies have demonstrated that glycocalyx production is also regulated by exposure to laminar shear stress^25^. In animals that received resuscitation with PolyHSA, the arteriole blood velocity and volumetric flow rate were substantially increased compared to the other two treatment groups. This effect is likely related to the increased circulatory half-life^17^ and molecular size of PolyHSA (24 h, and 410 kDa) compared to unmodified HSA (16 h and 67 kDa). These effects are likely the cause of the sustained volume expansion following fluid resuscitation with PolyHSA. Given that the viscosity of PolyHSA (4.1 cP) is significantly higher than unmodified HSA (1.5 cP), PolyHSA-enhanced plasma viscosity may have some effect on restoring vascular function due to additional wall shear stress.

One potential mechanism of decreased vascular retention after PolyHSA resuscitation was improved regeneration of the endothelial glycocalyx. Unfortunately, we were unable to observe the glycocalyx structure and shedding during these studies directly. Future studies should include a direct examination of the endothelial glycocalyx throughout treatment with the PolyHSA solution. In addition, the effect of other properties of PolyHSA, such as protein concentration and molecular weight, should be investigated.

### Limitations

LPS induced endotoxemia and CLP cannot fully replicate the events that occur during all cases of septic shock, given septic shock’s distinct etiologies. However, each model used herein represents a different facet of septic shock, and as such, demonstrates that PolyHSA is efficacious in a variety of conditions. Furthermore, anesthesia has poorly characterized effects on inflammation progression, which may confound results in the partially anesthetized CLP model. Future preclinical studies should examine the effectiveness of PolyHSA in other animal models to confirm that these effects are maintained. Additionally, this study did not examine parameters that directly measure the glycocalyx status, but instead examined the effects of PolyHSA on the glycocalyx’s primary physiological role: preservation of vascular permeability. Future studies should more directly examine glycocalyx integrity via fluorescent lectin binding or measure plasma changes in glycocalyx constituents during the progression of septic shock and resuscitation with PolyHSA.

## Conclusions

By using a controllable LPS inducible endotoxemia model and a more physiologically relevant CLP induced polymicrobial sepsis model, we found that resuscitation with PolyHSA significantly improves microvascular recovery from septic shock. Overall, the increased M.W. of PolyHSA compared to unmodified HSA played a critical role in maintaining microvascular hemodynamics by interrupting the positive feedback loop in endotoxemia, which stems from glycocalyx disruption. This study also demonstrates that the microvascular response to LPS induced endotoxemia presents more rapidly than macrovascular changes. As such, the development of techniques and instruments to more easily measure the function of microcirculation in the clinic could garner crucial diagnostic information and allow healthcare providers to react to the consequences of impaired microcirculatory function quickly.

Apart from microbial sepsis and LPS induced shock, this data suggests that PolyHSA resuscitation may have a role in the treatment of viral sepsis and secondary bacterial infection associated with COVID-19^26^. Infection from SARS-CoV-2 increases vascular permeability via angiotensin-converting enzyme 2 (ACE2) reduction, increases production of ROS by activated neutrophils, and initiates a cytokine storm^27^. A resuscitation fluid with reduced transport across damaged endothelium such as PolyHSA may be beneficial in reducing SARS-CoV-2 associated multiorgan failure.

## Methods

### Polymerized human serum albumin synthesis

The HSA (Albuminar^®^) used in this study was obtained from ABO Pharmaceuticals, San Diego, CA. Polymerization of HSA was performed as previously described^18^. In brief, HSA was incubated with glutaraldehyde at a 30:1 molar ratio of glutaraldehyde to HSA. The polymerization reaction was incubated for 3 h at 37 °C. The reaction was quenched with sodium borohydride (NaBH_4_). After the reaction, the resulting PolyHSA was diafiltered into a modified Ringer’s Lactate solution (115 mM NaCl, 4 mM KCl, 1.4 mM CaCl_2_, 13 mM NaOH, 27 mM sodium lactate, and 2 g/L N-acetyl-l-cysteine) on a 100 kDa polysulfone hollow fiber filter (Spectrum Labs, Rancho Dominguez, CA) for four diafiltration cycles. All PolyHSA samples were filtered through a 0.2 μm filter. The viscosity of unmodified HSA and PolyHSA solutions was measured with a DV-II + cone and plate viscometer (Brookfield Engineering Laboratories, Middleboro, MA) at a shear rate of 160 s^−1^^17, 18^. The COP of the solutions were measured with a 4420 membrane colloid osmometer (Wescor, Logan, UT).

### Animal preparation

All the procedures, including animal handling and care, followed the National Institutes of Health (NIH) Guide for the Care and Use of Laboratory Animals. The U.C. San Diego Institutional Animal Care and Use Committee approved the experimental protocol. All methods were carried out in accordance with the ARRIVE guidelines (Animal Research: Reporting of In Vivo Experiments). Mice and hamsters were fitted with a dorsal skinfold window chamber for direct visualization of the microcirculation. This model has been used widely to characterize the perfusion of peripheral tissues in unanesthetized animals, as previously described^28^. Briefly, animals were anesthetized with sodium pentobarbital (50 mg/kg i.p.), the dorsal area depilated, and the skinfold was lifted from the back using sutures. The skinfold was then captured between two titanium frames, each with a circular opening for visualization. The skin on one side of the window chamber was removed, following the outline of the circular window, and the exposed skin was covered with a glass coverslip. Two days after window chamber implantation, the mice and hamsters were anesthetized again, and a heparinized catheter was implanted in the left common carotid artery. Mice were then allowed 2 additional days for recovery before any experimental procedures were performed, and hamsters were immediately subjected to the CLP procedure, as described below.

### Endotoxemia protocol

Male Balb/c mice (23–28 g, Jackson Laboratory) were used for this experimental study. All animals were housed under the same conditions until the day of the experiment (12 h day/night cycles; approximately 25 °C and 60% humidity). Only animals within the defined inclusion criteria were used in this study. Baseline parameters were collected after acclimatizing to the experimental environment for at least 15 min. Animals received 10 mg/kg of lipopolysaccharide (LPS) from *E. coli* serotype 0128:B12 (Sigma, St. Louis, MO), suspended in 0.1 mL of saline via the arterial catheter. Fluid resuscitation was performed 1 h after LPS injection in the relevant groups and consisted of a single infusion of 30% of the animal’s blood volume (estimated as 7% of body weight) over 10 min. No additional therapies were given. Food and water were available ad libitum between observation time points. All animals survived the experimental protocol.

### Cecal ligation and puncture protocol

Male Golden Syrian Hamsters (50–70 g, Charles Rivers Laboratories) were used for this study. A state of polymicrobial sepsis was induced using the CLP model as described elsewhere^29, 30^. Briefly, animals were as described above. After shaving and disinfection of the animal’s abdomen, a 1 to 2 cm laparotomy was performed in the left flank and the cecum was exteriorized. Then, the cecum was ligated using a sterile silk suture and perforated near the base 2 times using a 20-gauge needle. Before replacing the cecum in the abdominal cavity, it was gently pressed to release part of the intestinal content. The laparotomy was closed, and the animal was left to recover in a 37 °C heating pad. To avoid excessive surgical interventions in a single animal, we performed the CLP procedure during the same surgical session used for catheter implantation. Fluid resuscitation was performed 1 h after CLP in the relevant groups and consisted of a single infusion of 30% of the animal’s blood volume (estimated as 7% of body weight) over 10 min. No additional therapies were given. All animals survived the initial CLP procedure. Microvascular and systemic monitoring of the animals began 1 h after full recovery from anesthesia. Continued survival was monitored for 4 days following the initial procedure.

### Inclusion criteria

Animals were considered suitable for the experiments if: (1) systemic parameters were within normal range at baseline. Namely, heart rate (HR) > 350 beats/min and mean arterial pressure (MAP) > 100 mmHg; and (2) microscopic examination of the tissue in the window chamber did not reveal signs of edema or bleeding under × 650 magnification.

### Experimental groups

Eighteen (18) animals were included in each arm (endotoxemia and polymicrobial sepsis) of this study and were divided into three experimental groups, named by the resuscitation fluid given (or lack thereof), namely: No resuscitation (no fluid resuscitation given, n = 6); HSA (human serum albumin at 10 g protein per dL fluid, n = 6); and PolyHSA (polymerized human serum albumin at 10 g protein per dL fluid, synthesized as described above, n = 6).

### Systemic parameters

MAP and HR were monitored continuously during the observation periods using the arterial line and a transducer-computer interface (MP150; Biopac Systems, Santa Barbara, CA).

### Microcirculatory hemodynamics

The window chamber was studied using transillumination on an upright microscope (BX51WI, Olympus, New Hyde Park, NY). Measurements were carried out using a 40× water immersion objective (LUMPFL-WIR, numerical aperture 0.8, Olympus). The microscope was equipped with a high-speed video camera (Fastcam 1024 PCI, Photron, USA), which was used to record videos of the microcirculation at 1000 frames per second. The animals were briefly restrained in a plexiglass tube with a longitudinal opening from which the window chamber protruded. Animals were then fixed to the stage of the microscope. Individual arterioles were identified at baseline based on visual clarity and followed throughout the experiment to improve statistical power. At each time point, a video recording of the individual vessels was captured and then analyzed offline as previously described^31^. The volumetric flow rate was estimated from the measured diameter (D) and centerline velocity (V) as $Q = \pi \times V \left( {D/2} \right)^{2}$. Shear stress was estimated from the measured values as $\tau = 8V/D$.

### Functional capillary density

Functional capillary density (FCD) was measured by counting the number of capillaries with a transit of at least a single red blood cell in a 45 s period. Ten consecutive microscopic visual fields are selected at baseline and monitored at different time points throughout the experiment.

### Tissue apoptosis and necrosis

Apoptotic and necrotic cells are labeled in situ by infusion of propidium iodide (P.I.) and Annexin V (0.14 mg each in 140 μL saline per animal; Molecular Probes, Eugene, OR). The dye was allowed to circulate for 30 min. Images were acquired using a high light-sensitive camera (C4742-95, Hamamatsu Photonics, Japan). A total of 40 microscopic fields were captured per animal, and the number of single-labeled and double-labeled cells were counted at each time point. Hair follicles and sebaceous glands were excluded from cell counts due to their consistently high rates of necrosis and apoptosis.

### Endothelial barrier permeability

Microvascular wall permeability was assessed by measuring the extravasation rate of fluorescein isothiocyanate conjugated dextran (FITC-Dextran; 70 kDa M.W.; Sigma, St. Louis, MO). The animals received a 100 μL bolus of FITC-Dextran (10 mg/mL) in the tail vein. The dye was allowed to circulate for 5 min, and locations of interest (containing arterioles, venules, and tissue) were selected prior to fluorescent imaging. The tissue was excited using a standard FITC filter cube, and images were recorded using a high light-sensitive camera (C4742-95, Hamamatsu Photonics, Japan). Images of the regions of interest were recorded at baseline and 6 h after LPS induction. A constant camera exposure time was used throughout the experiment. The images were analyzed offline by measuring the relative pixel intensity inside the microvessels (IV) and in the tissue adjacent to the microvessels (E.V.). Data were displayed as EV/IV, and high ratios indicate increased vascular permeability.

### Leukocyte endothelial interaction

To label leukocytes, animals received a 100 μL bolus of Rhodamine 6G (5 mg/kg; Sigma, St. Louis, MO) 5 min before the time point of interest. Fluorescently labeled leukocytes were excited, and images were captured with a Vivid Set (XF104-2 filter, Omega Filters, Brattleboro, VT) using a high-light sensitive camera (C4742-95, Hamamatsu Photonics, Japan). 60 s of video was captured on a straight portion of the vessel at 10 frames per second. During playback, the vessels were segmented into 100 μm lengths, and leukocytes were counted and classified as “rolling” or “adhered” to the endothelium as previously described^32^.

### Cytokine ELISA measurements

Plasma samples collected from animals at multiple time points were analyzed using a Multiplex Mice Cytokine ELISA Kit (R&D Systems, Minneapolis, MN) following the manufacturer’s instructions.

### Statistical analysis

Results are presented as mean ± standard deviation. All box plots are presented with the median on the centerline. The box limits are set to the upper (75%) and lower (25%) quartile. All outliers are shown in each plot. For all tests, P < 0.05 was considered statistically significant. Data analysis was performed in R (v 4.0.0) using the rstatix (v 0.3.1). Package. Data between groups were analyzed with a two-way Anova with Tukey’s test for post-hoc analysis. When possible, parameters were compared against baseline in the same animal or same vessel as a ratio relative to the baseline. For all tests, P < 0.05 was considered statistically significant. Survival data was analyzed with the survival (v 3.2.3) and survminer (v 0.4.7) packages.

### Ethics approval

All the procedures, including animal handling and care, followed the National Institutes of Health (NIH) Guide for the Care and Use of Laboratory Animals. The U.C. San Diego Institutional Animal Care and Use Committee approved the experimental protocol.

## Data Availability

The datasets generated during and/or analyzed during the current study are available from the corresponding author on reasonable request. For the original data, please contact pcabrales@ucsd.edu.
